# Electromagnetic Shielding Properties of Knitted Fabric Made from Polyamide Threads Coated with Silver

**DOI:** 10.3390/ma14051281

**Published:** 2021-03-08

**Authors:** Tanja Pušić, Bosiljka Šaravanja, Krešimir Malarić

**Affiliations:** 1 Department of Textile Chemistry and Ecology, Faculty of Textile Technology, University of Zagreb, 10000 Zagreb, Croatia; tanja.pusic@ttf.unizg.hr; 2 Department of Clothing Technology, Faculty of Textile Technology, University of Zagreb, 10000 Zagreb, Croatia; 3 Department of Communications and Space Technologies, Faculty of Electrical Engineering and Computing, University of Zagreb, 10000 Zagreb, Croatia; kresimir.malaric@fer.hr

**Keywords:** electroconductive material, polyamide, silver, shielding effectiveness, wet cleaning, dry cleaning

## Abstract

This paper investigates a textile material of low surface mass for its protection against electromagnetic radiation (EMR), which is suitable for composite structures of garments, and for technical and interior applications. The shielding effectiveness against EMR of fabric knitted from polyamide threads coated with silver, measured in the frequency range of 0.9 GHz to 2.4 GHz, indicated a high degree of protection. The key contribution of the paper is the evaluation of the stability of the shielding properties against EM radiation after applying apolar and polar solvents, in synergy with the cyclic process parameters of wet and dry cleaning. The results of the study confirmed the decline in the shielding effectiveness after successive cycles of material treatment with dry and wet cleaning. The effect of wet cleaning in relation to dry cleaning is more apparent, which is due to the damage of the silver coating on the polyamide threads in the knitted fabric.

## 1. Introduction

The increased awareness of EMR has led to the worldwide introduction of new regulations for manufacturers of electrical and electronic devices, which must now comply with the electromagnetic compatibility requirements (EMC requirements). The need to set limits for the EM radiation of electrical and electrical devices (mobile phones, microwave ovens, signals of ‘radar’ communication, radio transmitters etc.) that radiate EM energy in different frequency ranges aims to minimise the possibility of interference with radio and wired communications. The lifespan and efficiency of electronic devices can be increased by their protection against electromagnetic interference [1,2,3,4,5,6].

**Figure 1:**
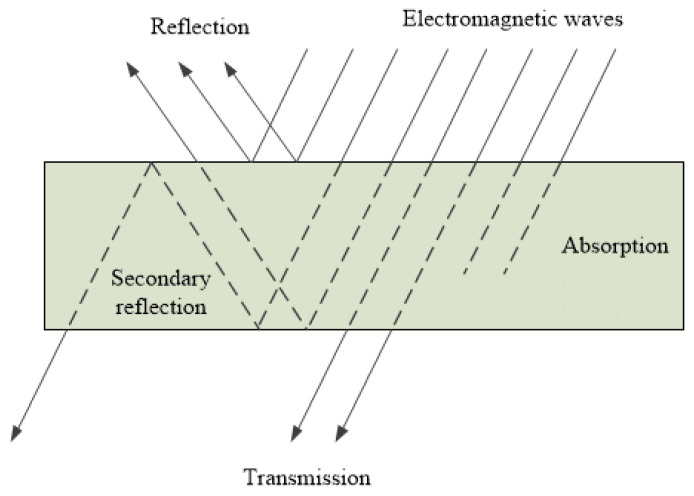
Schematic presentation of the signal propagation through a material with protective properties.

Figure 1 schematically shows the propagation of the signal through a layer of material with EMR protection properties. When EM rays pass through a medium or material, they interact with the molecules of the material; this phenomenon of interaction can be divided into three phases:

- absorption,
- reflection,
- secondary reflection.

When they hit the surface of a material, EM rays cause the charge in the material to oscillate. This forced oscillation of the charge acts as an antenna and results in reflection, whereas the other part is converted into thermal energy due to the oscillation. This kind of signal loss is known as attenuation due to absorption. Thus, the protective property of the material against EMR is based on the reflection against the conductive surface and the absorption in the conductive volume. Part of the wave is reflected, while the rest is transmitted and weakened as it passes through the medium [7].

The combined effect of losses through reflection and absorption determines the effectiveness of the protective properties of the material, depending on its electrical and magnetic properties, surface and interior conductivity properties, material thickness, material composition, abrasion, and degree of processing [8].

The ratio of the level of the electric field at a certain distance from the source without protection (shield) and the level of the electric field with protection is defined as the shielding effectiveness (*SE*). The shielding effectiveness of the conductive barrier *SE* in dB is the sum of loss of reflection (*R*), loss of absorption (*A*), and the loss of secondary reflection (*R_r_*), and is calculated according to Equation (1):

*SE* = *R* + *A* + *R_r_* (1)

For the purpose of protection against EMR in the electrical and electronic industry, conductive, lightweight and flexible textile structures are produced and developed instead of conductive metals or wire mesh materials. The reduction of the electromagnetic radiation transmission of textile materials can be achieved in various ways, such as by changing the composition [9], structure, or construction [10,11]; by the incorporation of conductive particles into the fibres, or metal threads and foils into the yarn [12,13,14]; or by the use of metal coatings [6,15], morphology [16], or conductive paints, pigments and varnishes [17].

Numerous studies of such products have been carried out, in which the different construction and finish parameters have varied. The results of measuring the protective properties of materials against EMR not only depend on the material properties but also on the sample size, measurement setup, and EMR source [1,18,19,20,21,22]. Since materials are exposed to various cyclic mechanical stresses, and chemical and atmospheric influences, it is important to monitor the durability of the protective properties under controlled conditions.

This paper deals with SE protective material made of silver-coated polyamide yarn, which—as a light and transparent structure—is suitable for applications in the composite structures of garments, and for technical and interior applications.

The functional material was analysed before and after the cyclic treatment in apolar and polar solvents, with process parameters of wet and dry cleaning. The influence of the solvents and process parameters on the changes on the surface of the material were analysed by scanning electron microscopy (SEM), while the protective properties of EMR were monitored by testing the properties of the shielding on the frequencies of 0.9 GHz, 1.8 GHz, 2.1 GHz, and 2.4 GHz.

## 2. Materials and Methods

The specifications of the shielding electrically-conductive knitted fabric made of polyamide (PA) yarn coated with silver (Ag) are presented in Table 1.

This functional knitted fabric can either be incorporated as a functional interlining in clothing or used to make children’s clothing due to its soft touch and antimicrobial properties, made possible by the silver. The non-stick type of interlining with protective properties against EM radiation is put between the base material and the lining, thus forming part of the composite structure of the garment. Garments are exposed to various mechanical and physicochemical influences, which makes it necessary to objectively evaluate the protective properties of their materials or composite structures before and after exposure to different frequencies. This is an important factor for the assessment of the service life of a garment with added value, which in this analysis is the protection against EMR (electromagnetic radiation).

The functional material (PA/Ag) is exposed to a cyclic treatment with a polar solvent (water) in wet cleaning, and an apolar solvent (perchloroethylene) in dry cleaning. These physicochemical processes were conducted through the synergy of solvents and the process parameters of the Sinner’s circle: chemistry, mechanical agitation, temperature, and time [23]. Wet cleaning (W) is an eco-friendly and under-researched process for SE textiles, which is conducted in water at a low temperature, with low mechanical agitation applying special hypoallergenic detergents and protective additives which reduce the swelling of fibres in water [24]. Dry cleaning (P) is a conventional process with excellent cleaning features, and is a promising basis for the retention of original material properties in perchloroethylene. PA/Ag knitted fabric with dimensions of 1 m × 1 m was treated with perchloroethylene 10 times, according to the norm EN ISO 3175-2, while the treatment with water was carried out according to the norm EN ISO 3175-3. The detailed specifications of the Sinner’s circle parameters in these processes are described in a piece of previously-published research [25].

### 2.1. Scanning Electron Microscopy (SEM)

The surface of the PA/Ag fabric was analysed before and after the cyclic treatment with an apolar solvent in dry cleaning (P) and a polar solvent in wet cleaning (W) under Sinner’s circle parameters; the samples were observed after the 1st, 3rd, 5th, 7th and 10th cycle. Despite the silver content in the PA/Ag fabric, all of the samples were coated with gold and palladium for 90 s using Emitech Mini sputter coater SC7620 (Quorum Technologies, Ashford, Kent, UK)). The observation of the samples’ surface was performed with the SE detector of the scanning electron microscope FE-SEM, MIRAIILMU, Tescan, Czech Republic, under a magnification of 500×.

### 2.2. Measuring the Shielding Effectiveness (SE) for Microwave Radiation

The shield properties of the tested samples were investigated using a method described in detail elsewhere [25], under the following working conditions:

- temperature 23 ± 1 °C,
- relative humidity 50 ± 10%.

**Figure 2:**
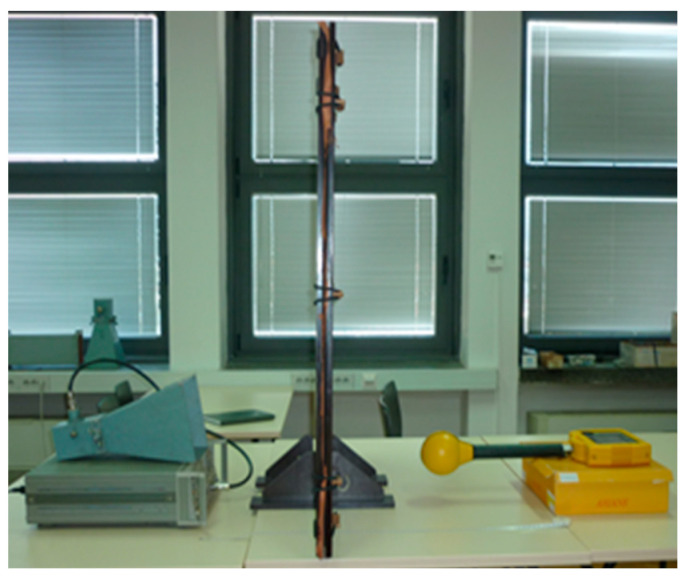
Measurement setup for the SE protective properties of textiles.

**Figure 3:**
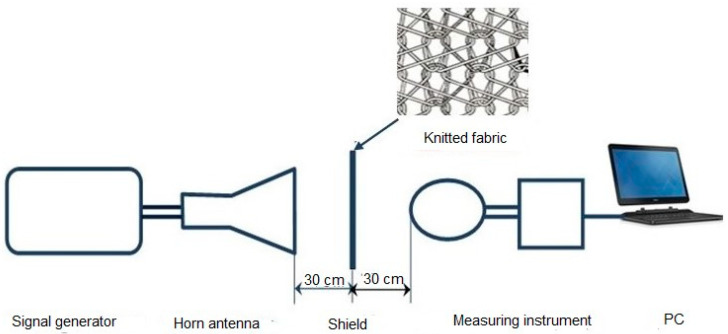
Schematic representation of the measurement setup.

According to the recommendations of the IEE-STD 299-97 [26], MIL STD 285 [27], and ASTM D-4935-89 [28], a measurement setup was designed and installed (Figure 2 and Figure 3), consisting of:

- a measuring instrument: NARDA SRM 3000,
- an HP 8350 B signal generator,
- an IEV horn antenna: Industrija za elektrozveze (Telecommunication Industry), Ljubljana, Type A12,
- a wooden frame, in which a sample of PA/Ag material of 1 m × 1 m was placed.

Figure 2 and Figure 3 show the measurement setup of the shield performance test. The signal generator was computer controlled, and provided frequencies of 0.9 GHz, 1.8 GHz, 2.1 GHz, and 2.4 GHz. The generator was connected to the horn/funnel antenna with a coaxial cable (for 900 MHz, a dipole antenna is used). The wooden shield was placed 30 cm away from the antenna and the measuring instrument: a spectrum analyser with a broadband antenna.

The EM protection factor was determined as the ratio between the EM field intensity (*E*_0_) measured without the fabric and the EM field intensity (*E*_1_) with the material placed between the radiation source and the measuring device.

The shielding effectiveness *SE* (dB) was calculated according to the following Equation (2):

(2) $$SE=20\log\frac{E_{0}}{E_{1}}$$

where:

- *E*_0_ is the field level without protection (shield),
- *E*_1_ is the field level with protection (shield).

The change in the shielding effectiveness of the PA/Ag knitted fabrics after the 1st, 3rd, 5th, 7th, and 10th cycles of dry and wet cleaning is expressed using Equations (3) and (4):

(3) $$\mathrm{dSE}={\mathrm{SE}}_{0}-{\mathrm{SE}}_{P}$$

(4) $$\mathrm{dSE}={\mathrm{SE}}_{0}-{\mathrm{SE}}_{W}$$

where:

- SE_0_ represents the initial shielding effectiveness of the PA/Ag knitted fabric,
- SE_P_ represents the shielding effectiveness of the PA/Ag knitted fabric after the 1st, 3rd, 5th, 7th, and 10th dry cleaning cycles,
- SE_W_ represents the shielding effectiveness of the PA/Ag knitted fabric after the 1st, 3rd, 5th, 7th, and 10th wet cleaning cycles.

## 3. Results and Discussion

The cyclic exposure of the PA/Ag material to solvents in synergy with the process parameters led to a change in the material thickness tested according to EN ISO 5084: 2003, as shown in Table 2.

Due to the presence of amide bonds in the macromolecules, PA fibres can form hydrogen bonds, owing to which they have a better ability to absorb moisture [29] (compared to some hydrophobic polymers), which implies the possible influence of the polar solvent, e.g., water (W). However, the results in the table indicate a slight increase in the thickness of the material in the wet cleaning (W) and dry cleaning (P) compared to the untreated fabric. Expressed in %, the variability of the fabric thickness after 10 dry cleaning cycles is 0.5 times higher than the variability of the fabric thickness after wet cleaning. The slight shrinkage of the SE fabric in the dry cleaning process can be explained by the presence of a small quantity of water in the system, and subsequent drying.

**Figure 4:**
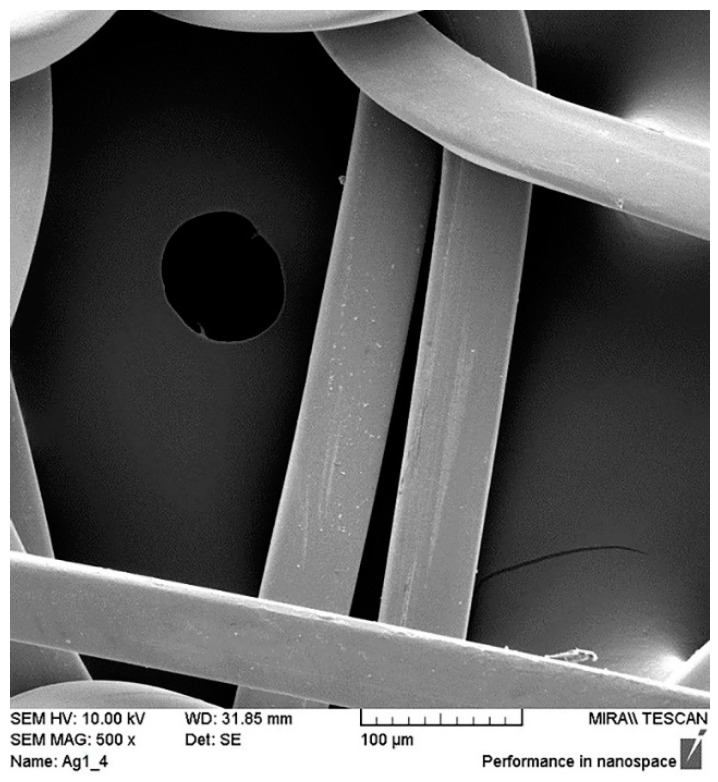
SEM micrograph of the conductive untreated PA/Ag sample under a magnification of 500×.

**Figure 5:**
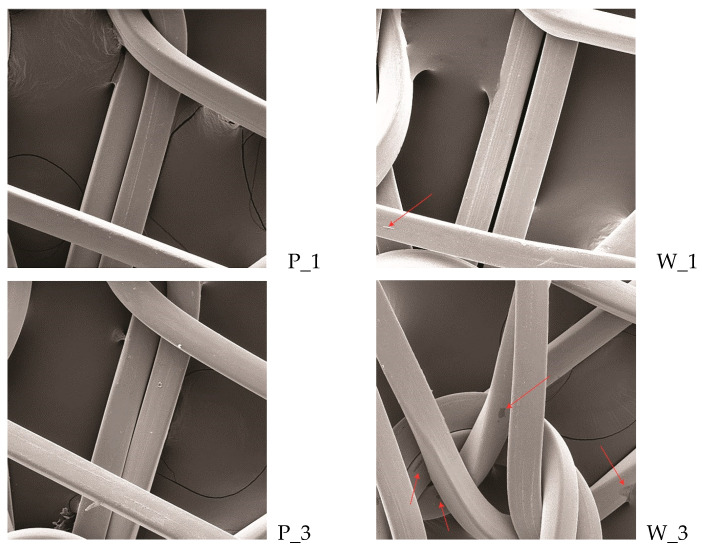

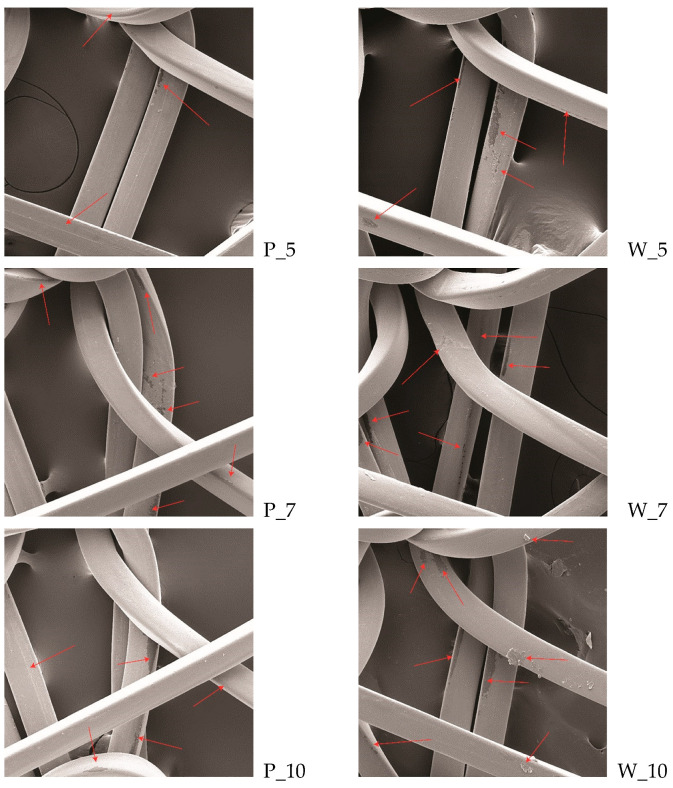
SEM micrographs of the PA/Ag samples before and after the 1st, 3rd, 5th, 7th and 10th treatment cycles of dry (P) and wet cleaning (W), under a magnification of 500×.

The photograph, shown in Figure 4, of the surface of the conductive untreated PA/Ag sample shows a uniform coating of silver on the polyamide filament. The synergistic influence of the solvent and other process parameters was achieved through the characterisation of the surface of the PA/Ag material by the scanning electron microscope before and after the 1st, 3rd, 5th, 7th, and 10th processing cycle, under a magnification of 500× (Figure 5).

The influence of the apolar solvent (P) in synergy with the dry cleaning process parameters could be seen after five cycles, which can be attributed to the successive cycles. With the increase in the number of cycles on the knitted fabric, greater longitudinal damage of the silver coating on the threads was noticed, which intensified in the 10th cycle. The irregular shape of the damage and the appearance of ruptures on the silver coating indicate a more intense influence of mechanics as a process factor, which led to fractures of the material. Such local damage excludes the influence of solvents, which would act more evenly over the entire surface. The changes in the surface of the PA/Ag fabric under the influence of the polar solvent (W) and wet cleaning process parameters were visible after the 3rd cycle. The change dynamics were more intense compared to the dry cleaning (P). Additionally, irregular incrustations could be seen on the sample surface after 10 cycles of wet cleaning (W_10), which indicate the interaction of some of the substances in the process. The SEM images indicate that the polar solvent, in synergy with the process parameters of wet cleaning, caused a higher degree of longitudinal and irregular local damage to the PA/Ag fabric compared to the apolar solvent and the process parameters of dry cleaning.

The examination of the durability of the protective properties of elastic yarns with metal coatings has confirmed that the changes in the conductive properties during washing depend on the type of coating. The obtained results deviate from the research conducted on silver-coated textiles after washing in 25 cycles; the silver coating remained almost unchanged [30].

The polyamide, as a pure polymer, exhibits non-conductive properties, while the coating with Ag enhanced the electrical conductivity of the material and increased its shielding effectiveness [31].

**Figure 6:**
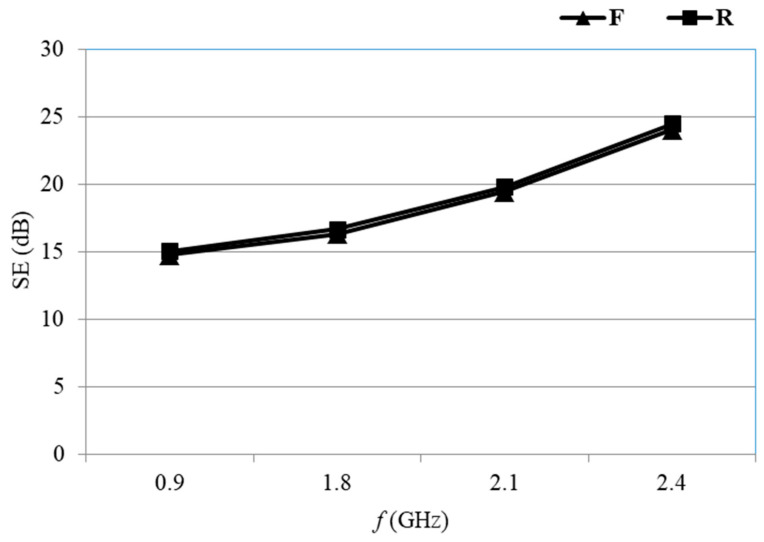
Initial SE of the face (F) and reverse (R) sides of the PA/Ag fabric at frequencies of 0.9 GHz, 1.8 GHz, 2.1 GHz, and 2.4 GHz.

The shielding effectiveness (SE) of the face and reverse side of the PA/Ag fabric before the solvent treatment with process parameters at frequencies of 0.9 GHz, 1.8 GHz, 2.1 GHz, and 2.4 GHz are shown in Figure 6.

The protective properties of the face and reverse side of the untreated PA/Ag samples at all of the frequencies are almost identical, as shown in Figure 6. The highest degree of protection was obtained at 2.4 GHz (24.1 dB), while the lowest degree of protection was achieved at 0.9 GHz (SE = 14.8 dB). Despite the difference of almost 10 units, the achieved degree of protection >10 dB represents an acceptable degree of protection [31].

**Figure 7:**
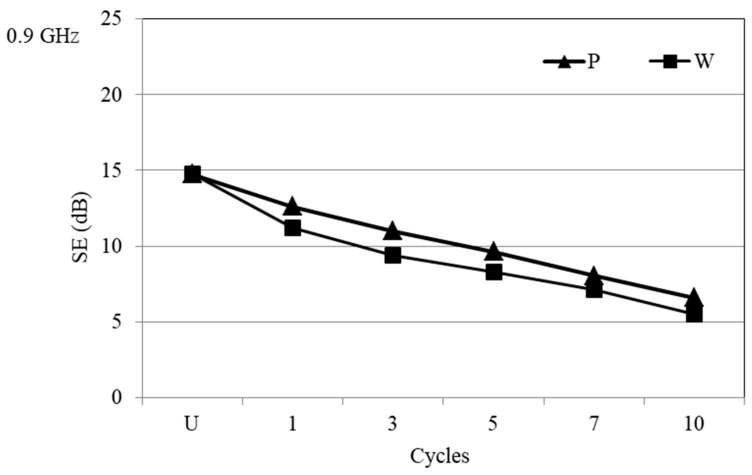
SE of the PA/Ag fabric before and after 10 cycles of treatment with P and W at the frequency of 0.9 GHz.

The first cycle of the PA/Ag fabric treatment with the dry cleaning (P) and wet cleaning (W) reduced the degree of protection at 0.9 GHz. The wet cleaning (W) had a stronger influence compared to the dry cleaning (P), while the largest difference of SE properties was confirmed after 3 cycles. The almost linear and parallel decline of the SE properties continues after the 5th, 7th, and 10th cycle (Figure 7).

**Figure 8:**
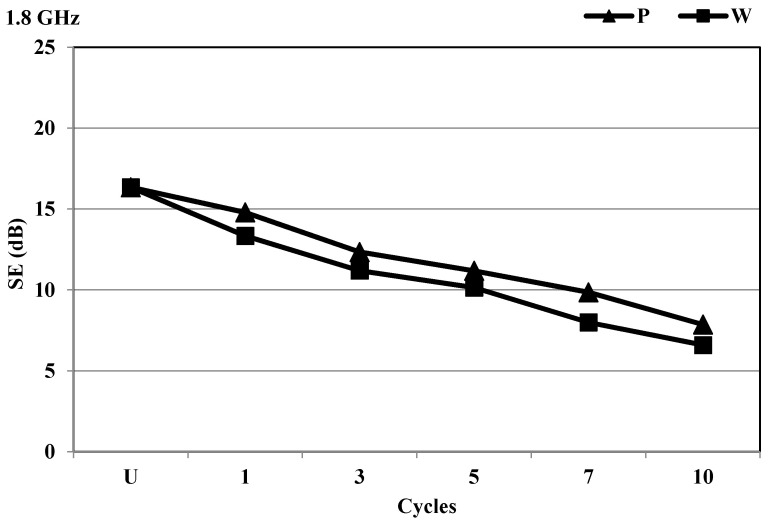
SE of the PA/Ag fabric before and after 10 cycles of treatment with P and W at the frequency of 1.8 GHz.

The first cycle of the PA/Ag fabric treatment with the dry cleaning (P) and wet cleaning (W) solvents, in synergy with process parameters, reduced the degree of protection at 1.8 GHz. The wet cleaning had a stronger influence compared to the dry cleaning; the largest difference of SE properties was found after the 7th cycle (Figure 8).

**Figure 9:**
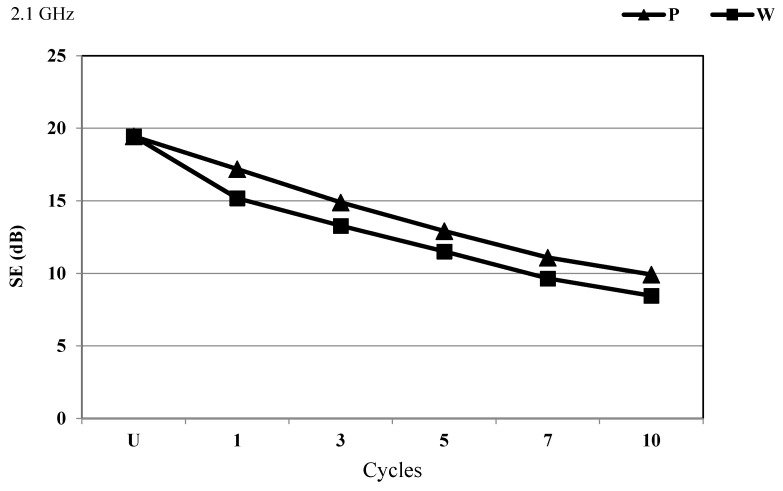
SE of the PA/Ag fabric before and after 10 cycles of treatment with P and W at the frequency of 2.1 GHz.

The first cycle of the PA/Ag fabric dry cleaning and wet cleaning reduced the degree of protection at 2.1 GHz. The wet cleaning had a stronger influence compared to the dry cleaning. The largest difference in SE properties was found after the 1st cycle, and the almost linear and parallel decline in the SE properties continued after the 3rd, 5th, 7th, and 10th cycle (Figure 9).

**Figure 10:**
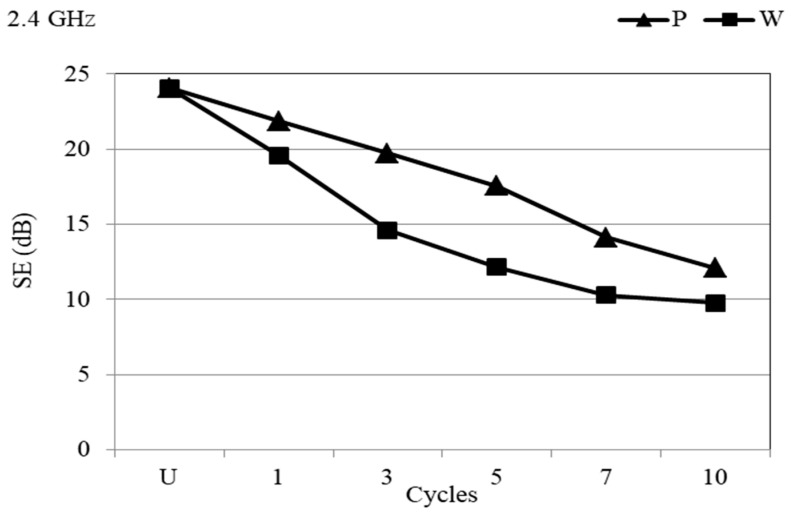
SE of the PA/Ag fabric before and after 10 cycles of treatment with P and W at the frequency of 2.4 GHz.

Textile materials characterized by a shielding effectiveness (SE) of >20 dB are acceptable for industrial applications [31], meaning that untreated PA/Ag fabric possesses an appropriate SE at a frequency of 2.4 GHz. Figure 10 indicates the better preservation of SE in dry cleaning than in wet cleaning. The numerical differences in the SE values of the PA/Ag fabric due to repeated processing cycles are shown in Table 3.

The SE differences of the treated PA/Ag fabric caused by the physicochemical influence of dry and wet cleaning in relation to the untreated fabrics are shown in Table 3 and Table 4.

Based on the obtained efficiency differences (dSE) at all frequencies, a more progressive influence of the wet cleaning on the decrease of the SE value compared to the dry cleaning could be clearly noticed. The largest efficiency differences were found at 2.4 GHz. The impact of the process parameters in wet and dry cleaning on dSE correlated well with the surface observation of the SEM images.

The initial surface damage of PA/Ag fabrics is observed after the 5th dry cleaning. The obtained dSE values of the PA/Ag fabric through five cycles of dry cleaning at all frequencies are almost equal. The differences in dSE between the lower and higher frequencies are noticeable after the 7th and 10th cycles of dry cleaning.

The subsequent cycles of wet cleaning affect larger differences at all frequencies compared with the 1st cycle. The initial surface damage of the Ag layer on the PA fabric observed after the 3rd wet cleaning cycle caused a great decrease in the dSE value, especially at a frequency of 2.4 GHz.

It has been confirmed that the damage of the fabric surface layer in the wet cleaning process affects the decrease in SE values. The obtained results for the samples treated in the wet cleaning process were not in accordance with the results presented in [32], in which the Electromagnetic Shielding Effectiveness (EMSE) values in a low and medium frequency range (0.75 GHz to 3.0 GHz) were attributed to changes in the fabric structure after five cycles of the washing process.

## 4. Conclusions

Polyamide knitted fabric made of silver-coated thread possesses an optimal electromagnetic shielding effectiveness in the frequency range from 0.8 GHz to 2.4. GHz. The protective factor, minimal weight and thickness are promising characteristics for clothing, interior, and technical applications. The initial SE properties were changed under repeated cycles of dry and wet cleaning. The increased number of wet and dry cleaning cycles caused a linear drop of the SE values at 0.9 GHz, 1.8 GHz, 2.1 GHz, and 2.4 GHz. The SEM images indicated the damage of the silver coating on the polyamide yarn. The degradation was more noticeable after the wet cleaning than the dry cleaning.

## Figures and Tables

**Table 1:** Specifications of the shielding conductive knitted fabric (PA/Ag).

Composition PA/Ag (%)	80/20	
Mass per unit area (g/m^2^)	35.8	
Density (course/wale)/100 mm	150/125	
Charmeuse knitted structure	digital microsope images	
	magnification 50×	magnification 250×
	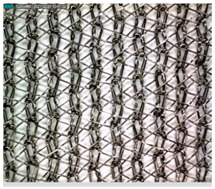	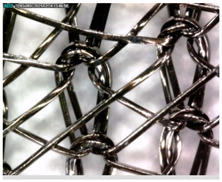

**Table 2:** Thickness of the PA/Ag fabric before and after 10 cycles of dry (P) and wet (W) cleaning.

PA/Ag Fabric	Thickness (mm)
Untreated	0.150
10 cycles treatment with P	0.162
10 cycles treatment with W	0.165

**Table 3:** Difference in the shielding effectiveness of the PA/Ag fabric after the impact of dry cleaning (P).

*f* (GHz)	Dry Cleaning Cycles				
	dSE (dB)				
	P_1	P_3	P_5	P_7	P_10
0.9	2.2	3.8	5.2	6.7	8.2
1.8	1.5	4.0	5.2	6.5	8.5
2.1	2.3	4.6	6.5	8.4	9.5
2.4	2.2	4.3	6.5	9.9	11.9

**Table 4:** Difference in the shielding effectiveness of the PA/Ag fabric after the impact of wet cleaning (W).

*f* (GHz)	Wet Cleaning Cycles				
	dSE (dB)				
	W_1	W_3	W_5	W_7	W_10
0.9	3.6	1.8	6.4	7.6	9.3
1.8	4.3	6.2	7.9	9.8	10.9
2.1	4.3	6.2	7.9	9.8	10.9
2.4	4.5	9.5	11.9	13.8	14.3

## Data Availability

Data available in a publicly accessible repository.
